# CW‐Pumped Amplified Spontaneous Emission From Thermally Evaporated CsPbBr_3_ Films

**DOI:** 10.1002/advs.78165

**Published:** 2026-10-11

**Authors:** Yuliia Kominko, Willem Verheijen, Gabriele Rainò, Simon C. Boehme, Maksym V. Kovalenko, Sergii Yakunin

**Affiliations:** ^1^ Department of Chemistry and Applied Biosciences Laboratory of Inorganic Chemistry ETH Zürich Zürich Switzerland; ^2^ Empa – Swiss Federal Laboratories for Materials Science and Technology Laboratory For Thin Films and Photovoltaics Dübendorf Switzerland

**Keywords:** amplified spontaneous emission, cesium lead bromide, continuous‐wave excitation, thermal degradation, thermally evaporated films

## Abstract

Lead halide perovskites are widely reported as promising optical gain media for optoelectronic applications in the short‐pulse regime. However, their operation under continuous‐wave excitation remains severely limited by thermal accumulation and gain instability. Here, we present a systematic study of amplified spontaneous emission under long‐pulse and continuous‐wave excitation in thermally evaporated CsPbBr_3_ thin films, providing direct insight into the limits of sustained optical gain in perovskite materials. By correlating excitation pulse duration, substrate thermal conductivity, temperature‐dependent emission properties, and time‐resolved carrier dynamics, we establish a physical framework linking thermal dissipation to gain stability. We identify excitation‐time thresholds and define optimal operating regimes, demonstrating sustained amplified spontaneous emission for pulse widths up to 30 µs in films without encapsulation or additional post‐processing. Our results reveal that light‐induced thermal accumulation is the dominant mechanism limiting amplified spontaneous emission under extended excitation and highlight thermal management as a critical design parameter for steady‐state optical gain. These findings provide practical design rules for perovskite gain media and represent a key step toward electrically pumped, continuous‐wave‐performing perovskite superluminescent and laser diodes.

## Graphical abstract

Thermally evaporated CsPbBr_3_ thin films sustain amplified spontaneous emission from short‐pulse toward quasi‐continuous‐wave excitation. Time‐resolved spectroscopy, controlled excitation, and substrate engineering reveal how excitation duration governs optical‐gain stability. High‐thermal‐conductivity sapphire extends stable ASE to longer excitation pulses, highlighting thermal management as a key requirement for sustained perovskite optical gain.

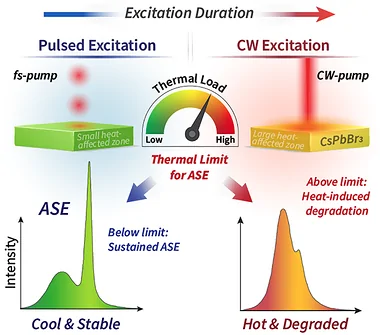

**Abbreviations**
AFM: atomic force microscopy
ASE: amplified spontaneous emission
CW: continuous‐wave
EDS: energy‐dispersive spectroscopy
FFT: fast Fourier transform
FWHM: full width at half‐maximum
LHPs: lead halide perovskites
NCM: non‐contact mode
PL: photoluminescence
RT: room temperature
SEM: scanning electron microscopy
TRPL: time‐resolved photoluminescence
WGM: whispering‐gallery mode

## 1 Introduction

Lead halide perovskites (LHPs) have emerged as a promising class of semiconductor materials due to their exceptional optoelectronic properties. They can be synthesized in a scalable and cost‐effective manner at low temperatures and pressures, often under ambient conditions. LHPs exhibit broad spectral tunability across the visible range, narrow emission linewidths, high absorption coefficients, long charge‐carrier diffusion lengths and lifetimes, and a high degree of defect tolerance [1, 2, 3, 4, 5, 6, 7]. Beyond their success in photovoltaic and light‐emitting applications, the outstanding optical gain properties of LHPs have positioned them as highly promising materials for amplified spontaneous emission (ASE) and lasing [8, 9, 10, 11, 12, 13, 14].

In particular, the combination of high absorption coefficients, long carrier lifetimes, large oscillator strength, and defect tolerance enables efficient population inversion and low‐threshold stimulated emission in perovskite gain media [15, 16, 17, 18]. However, achieving reliable ASE under continuous‐wave (CW) excitation remains a critical milestone toward practical perovskite‐based lasers, as CW operation requires sustained optical gain under steady‐state excitation. In contrast to pulsed excitation, CW pumping leads to continuous carrier generation and pronounced heat accumulation, which can result in enhanced nonradiative Auger recombination and, ultimately, material degradation [19, 20, 21, 22]. Thermal effects are especially critical in perovskite thin films, where limited heat dissipation can significantly affect emission stability and gain performance [8]. Consequently, despite rapid progress in perovskite gain media, CW‐pumped ASE has remained limited to rare demonstrations under highly restrictive conditions, such as quasi‐CW excitation or cavity‐enhanced configurations [23, 24, 25]. To date, stable ASE from perovskites under CW excitation has been demonstrated only by Lemmer et al. in phase‐stable CH_3_NH_3_PbI_3_, with an excitation intensity of 720 W cm^−2^ at 80 K [19]. These results underscore the prospects of perovskite gain media and highlight the need for a thorough understanding of the interplay between optical gain and thermally induced effects in CW‐pumped ASE in perovskites, which remains lacking.

In our previous work, we demonstrated that improved thermal management enables single‐source thermally evaporated LHP thin films to exhibit outstanding operational stability, high optical gain, and tunable low‐threshold ASE under femtosecond‐laser excitation [8]. Building on these findings, we present advanced studies of long‐pulse‐ and CW‐pumped ASE specifically in CsPbBr_3_ films evaporated on high‐thermal‐conductivity substrates. We show that the key distinction between pulsed and CW excitation lies in their thermal dynamics: short‐pulse excitation preserves optical gain through limited heat accumulation and efficient thermal relaxation, whereas long‐pulse and CW excitation are fundamentally constrained by light‐induced thermal buildup, which suppresses and ultimately degrades ASE. We used multiple excitation pulses and beam experimental methods, combining spatial and temporal overlaps of the pulses/beams to characterize excitation duration, thermal dissipation, and gain dynamics. Through these experiments, we directly identify the thermal limits for sustained ASE under extended excitation, the excitation‐time thresholds for stable ASE, and define optimal operating regimes for thermally evaporated CsPbBr_3_ films on sapphire substrates with pulse widths up to 30 µs. Furthermore, our results provide strong evidence that thermal accumulation is the dominant factor limiting ASE under long‐pulse and CW excitation, and highlight thermal management as a critical design parameter for steady‐state optical gain, addressing a major obstacle to realizing practical electrically pumped and CW‐operated perovskite lasers.

## 2 Results and Discussion

### 2.1 Origin of Amplified Spontaneous Emission From Thermally Evaporated CsPbBr_3_ Films

The perovskite thin films investigated in this work were fabricated by the single‐source thermal evaporation of melt‐grown CsPbBr_3_, as described in our previous publication [8]. The resulting films exhibit a compact nanocrystalline morphology with high structural uniformity, promoting efficient thermal dissipation and stable optical gain. The absorption spectrum exhibits a pronounced excitonic feature at 512 nm, associated with the formation of nanocrystalline grains within the evaporated film (Figure 1a and Figure S1). At room temperature, the thermally evaporated CsPbBr_3_ films show green photoluminescence (PL) with a peak at approximately 522 nm, along with a low‐threshold ASE band emerging near 544 nm under femtosecond‐pulse excitation. The spectral overlap between the PL band and the low‐energy tail of the absorption spectrum indicates that spontaneously emitted photons can stimulate further emission via the same optical transitions, making this overlap a key indicator of optical gain potential and population inversion (the fundamental requirement for stimulated emission). Moreover, due to the exceptional properties of thermally evaporated CsPbBr_3_ films, a pronounced ASE band near 533 nm was observed under 450 nm CW excitation in a nitrogen atmosphere at 90 K (Figure 1b). Below the ASE threshold of 2.07 W (corresponding to an excitation density of 45 kW cm^−2^ for a 130 × 35 µm^2^ beam size), only spontaneous emission was observed (Figure 1b, inset).

**FIGURE 1:**
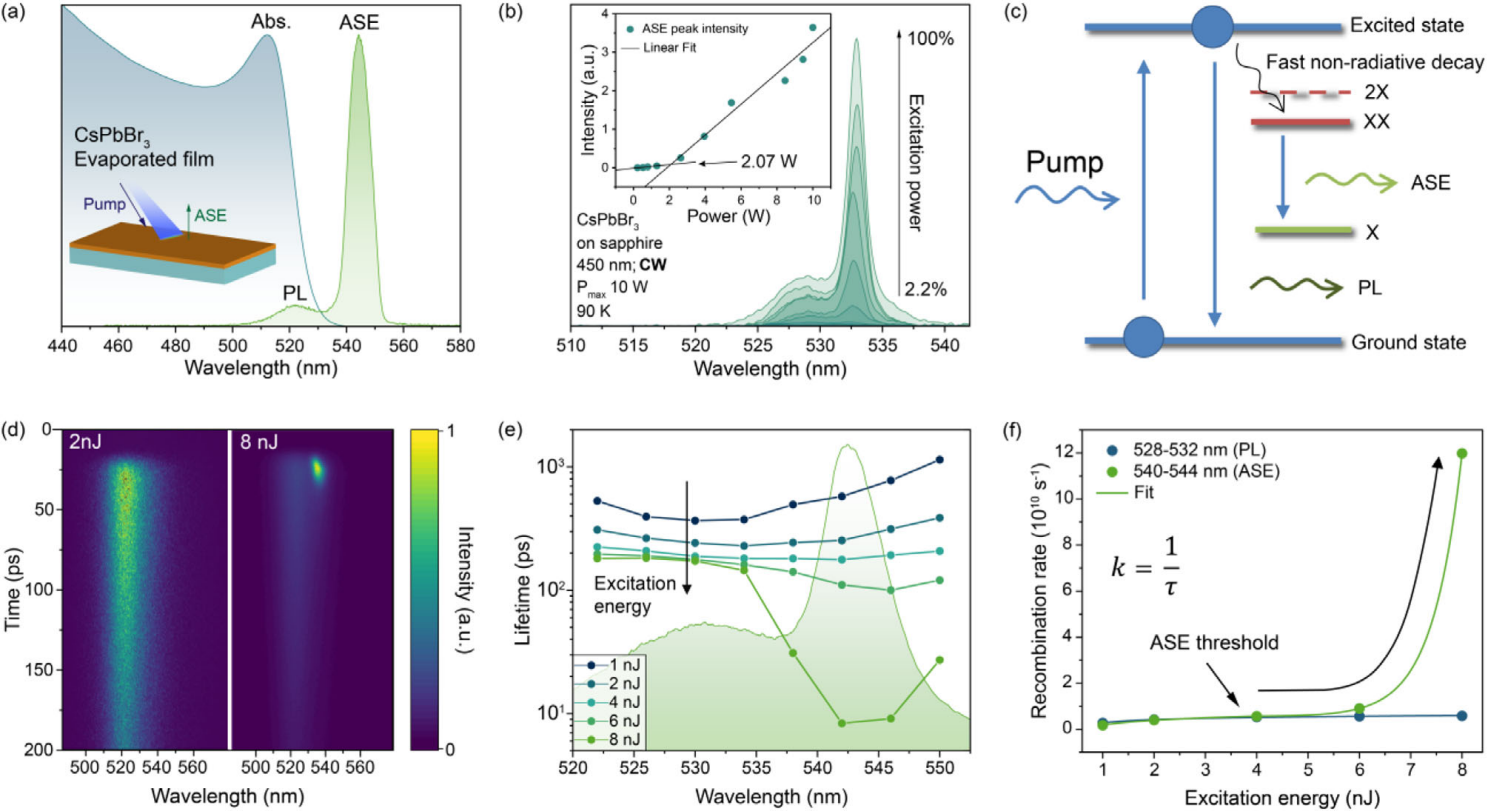
Amplified spontaneous emission from a 100‐nm‐thick thermally evaporated CsPbBr_3_ film. (a) Normalized absorption (blue curve) and emission (green curve) spectra of the evaporated film on a soda‐lime glass substrate with 250 fs‐laser excitation at 450 nm, 10 kHz repetition rate, and 6.2 µJ cm^−2^ energy density at room temperature (RT). (b) Emission spectra of the evaporated film on a sapphire substrate at different excitation powers of the 450‐nm CW laser at 90 K. Inset: emission intensity at the ASE peak wavelength (near 533 nm) vs. excitation power. The ASE threshold was determined to be 2.07 W (45 kW cm^−2^ for a 130 × 35 µm^2^ beam size) from the intercepts of the linear fits (black line) below and above the threshold. (c) Energy diagrams of the biexciton model for ASE and spontaneous emission from the thermally evaporated CsPbBr_3_ film. (d) Time‐ and wavelength‐resolved streak camera images under 450 nm fs‐laser excitation (excitation of 2 nJ, left, and 8 nJ, right, with 10 kHz repetition rate) at RT. (e) Corresponding wavelength‐dependent fitted lifetimes at different excitation energies, together with the emission spectrum under 8 nJ excitation energy (green curve on the background). (f) Corresponding recombination rate vs. excitation energy in the PL region (spectrally integrated between 528 and 532 nm; dark blue marks) and ASE region (spectrally integrated between 540 and 544 nm; green marks), demonstrating ASE threshold at 4 nJ.

In principle, several mechanisms can contribute to stimulated emission in LHPs, including excitonic‐stimulated emission, biexciton recombination, electron‐hole plasma recombination, band filling, and trap‐assisted stimulated emission [26, 27]. Among these mechanisms, we consider biexciton‐mediated gain the most plausible in our system [28, 29, 30]. To further examine this hypothesis, we performed excitation‐dependent time‐resolved photoluminescence (TRPL) measurements over a broad range of excitation intensities (Figure S2). At high excitation densities, an additional fast decay component becomes clearly resolved in the TRPL dynamics. The emergence and increasing contribution of this fast component with increasing excitation density are consistent with biexciton‐related recombination and therefore provide additional support for a biexcitonic contribution to the optical gain [31, 32]. Although our observations provide robust support for a biexciton‐mediated contribution to optical gain, other carrier‐density‐dependent processes cannot be fully excluded. The biexciton‐mediated gain mechanism is schematically illustrated in Figure 1c. Under an optical pump, electrons are excited from the valence band to the conduction band, generating electron‐hole pairs. These carriers rapidly relax via nonradiative processes into lower‐energy emissive states. At sufficiently high excitation densities, carrier accumulation leads to the formation of biexcitonic (XX) or multi‐excitonic states, which act as the primary gain reservoir and enable population inversion. Radiative recombination from these states gives rise to ASE when stimulated emission dominates over absorption. At low excitation densities, carriers recombine radiatively from the single‐exciton (X) state, producing spontaneous emission.

Time‐ and wavelength‐resolved streak camera measurements performed at various femtosecond excitation energies provide direct insight into the emission dynamics (Figure 1d and Figure S3). At low excitation energy (2 nJ), the emission is dominated by spectrally broad and long‐lived spontaneous emission. In contrast, at higher excitation energy (8 nJ), a redshifted, spectrally narrow emission band emerges, along with a much faster decay. By fitting the slow and fast decay components associated with PL and ASE, respectively, we observe that both the ASE decay and the PL lifetime increase as the excitation energy is reduced (Figure 1e). While the ASE lifetime is estimated to be shorter than 10 ps, excitation pulses that substantially exceed this timescale provide effectively continuous carrier generation on the timescale of ASE dynamics. However, they should be distinguished from true CW excitation with respect to the thermal response of the system.

Additionally, analysis of recombination rates vs. excitation energy reveals clear threshold behavior in the ASE spectral region (540–544 nm), whereas the PL region (528–532 nm) shows much weaker dependence on excitation energy (Figure 1f). This distinct nonlinear increase in the recombination rate at the ASE wavelength provides strong evidence for stimulated emission. Taken together, our experimental data indicate that the most plausible origin of ASE in thermally evaporated CsPbBr_3_ films is specifically a biexciton‐mediated mechanism.

### 2.2 Impact of Long‐Pulse Excitation on Amplified Spontaneous Emission

Optical amplification, or ASE, requires a population inversion induced by sufficiently high excitation density. Under short‐pulse excitation, carriers are injected rapidly at very high peak power but with relatively low average power. In addition, sufficiently long intervals between short excitation pulses allow thermal relaxation of the gain medium, thereby limiting cumulative heat accumulation and helping to preserve optical gain (Figure 2a).

**FIGURE 2:**
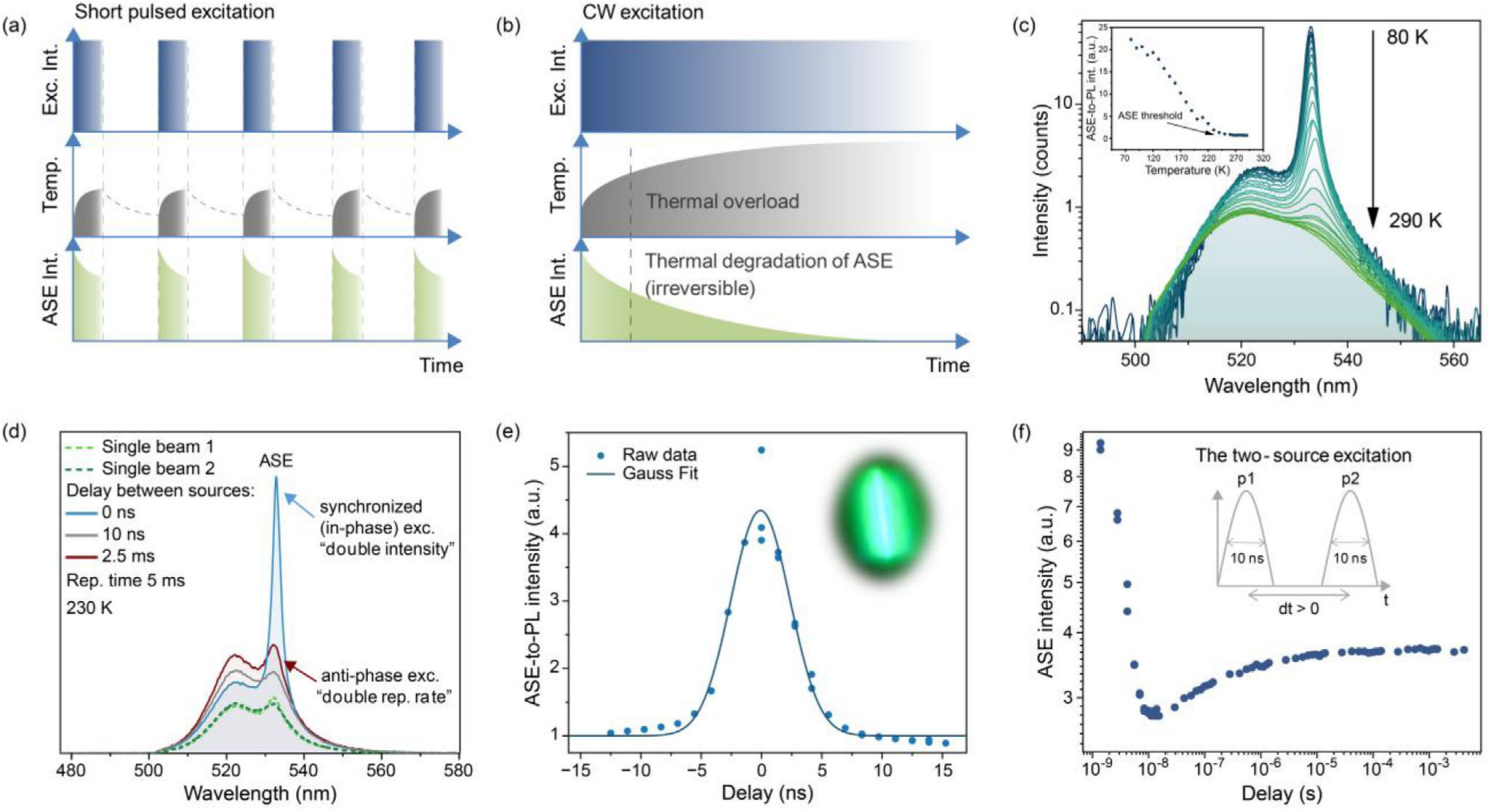
ASE from a 100‐nm‐thick thermally evaporated CsPbBr_3_ film on soda‐lime glass under 10‐ns pulsed excitation. (a,b) Schematic graph of time‐resolved temperature and ASE intensity under short‐pulsed (a) and CW excitation (b), showing light‐induced thermal degradation of the ASE under long‐pulsed (or CW) excitation. (c) Temperature‐dependent emission spectra with ASE band from thermally evaporated film under 10 ns‐laser excitation (450 nm, 1 kHz, 360 nJ or 3 mJ cm^−2^ for 240 × 50 µm^2^ beam size). Inset: ASE‐to‐PL intensity ratio vs. temperature showing threshold behavior of ASE. (d) Emission spectra with ASE band from thermally evaporated film under excitation from two beams of 10 ns‐lasers with 0 ns (blue curve), 10 ns (grey curve) and 2.5 ms (dark red curve) delay between laser pulses (10 ns, 450 nm, 200 Hz, and 180 nJ each (or 1.5 mJ cm^−2^ for 240 × 50 µm^2^ beam size); beam spots are overlapped on the sample surface) at 230 K. (e) ASE‐to‐PL intensity ratio vs. delay between the two pulses together with the Gaussian fit of the experimental data. Inset: optical image of the two laser beams spatially overlapped on the sample surface. (f) Corresponding ASE intensity vs. delay between the laser pulses, demonstrating the maximum of light‐induced thermal degradation of ASE (i.e., minimum of ASE intensity) when the delay is 10 ns. All measurements were conducted in a nitrogen chamber.

In contrast, long‐pulse or CW excitation is a much more demanding regime for ASE. Under constant excitation, carriers are continuously generated, resulting in a substantially higher thermal load. Without recovery intervals, heat dissipates less efficiently, causing a progressive temperature rise in the gain medium (Figure 2b). Elevated temperatures enhance nonradiative recombination and suppress population inversion, thereby strongly reducing optical gain (Figure 2c). Additionally, continuous thermal accumulation may cause thermal overload and, consequently, local defect formation, followed by irreversible material degradation (Figure S4).

To gain deeper insight into optical‐gain behavior under long‐pulse excitation, we employed a two‐beam excitation regime using two identical 10 ns laser diodes at 450 nm, first focused onto the same spot on the sample surface to ensure full spatial overlap. Both laser diodes were controlled by separate channels of the functional generator, allowing us to carefully control temporal delays between the laser pulses. This approach enables us to investigate the interplay between temporal pulse overlap, carrier‐density‐dependent optical gain, and slower relaxation processes in the ASE response. When operated individually, two PL spectra with a weak ASE band are observed from the thermally evaporated CsPbBr_3_ film at 230 K (Figure 2d, green lines), indicating that the excitation density provided by each pump source is equal and slightly above the ASE threshold.

Meanwhile, when the two pulses arrive simultaneously, their temporal overlap approximately doubles the instantaneous excitation density. Since ASE exhibits a strongly nonlinear dependence on excitation density near threshold, this increased instantaneous carrier generation produces a pronounced enhancement of the ASE band (Figure 2d, blue line). In contrast, when the excitation pulses are sufficiently separated in time, the instantaneous excitation density approaches that of a single excitation source, and no comparable ASE enhancement is observed (Figure 2d, gray line). It reveals a minimum of the ASE feature at a time delay of 10 ns. This indicates that the excitation effectively corresponds to a longer integration time rather than an increase in instantaneous excitation density. For a 10 ns pulse excitation and a 200 Hz repetition rate, the system has sufficient time to thermally relax between excitation pulses. Notably, we selected 230 K based on the excitation energy available from the 10 ns laser diodes. As mentioned above, under excitation with a single 180 nJ pulse (1.5 mJ cm^−2^ for 240 × 50 µm^2^ beam size), the film exhibits predominantly spontaneous emission with only a weak ASE contribution, whereas temporal overlap of the two pulses results in a pronounced ASE band. This condition therefore provides sufficient sensitivity to probe changes in optical gain as a function of the relative pulse delay.

Analysis of the ASE intensity dependence on the temporal delay between the two excitation pulses reveals a pronounced minimum at approximately 10 ns (Figure 2e,f). For pulse delays shorter than 10 ns, two excitation pulses partially overlap in time. In this regime, the ASE response is strongly influenced by the increased instantaneous excitation density and the resulting nonlinear gain enhancement. As the delay increases, the temporal overlap decreases, reducing the peak carrier density and, consequently, the ASE intensity. At the same time, residual carrier populations and the onset of excitation‐induced heating may also contribute to the observed response. Therefore, the short‐delay behavior cannot be attributed exclusively to thermal effects.

At the same time, Gaussian fitting of the ASE‐to‐PL intensity ratio as a function of the temporal delay between excitation pulses yields a full width at half‐maximum (FWHM) of 5.8 ns. This autocorrelation reflects the temporal overlap of the excitation pulses coupled with the nonlinear ASE response. However, this specific time value cannot be interpreted as a characteristic thermal‐relaxation time. The thermal estimate in Note S1 further shows that through‐film thermalization occurs on a timescale of several tens of nanoseconds, whereas substantial heat dissipation into the substrate develops on the sub‐microsecond to microsecond scale. Thus, the ∼10 ns feature is primarily associated with pulse overlap and nonlinear carrier‐density‐dependent gain, while the recovery at longer delays is consistent with progressively increasing thermal relaxation.

Additionally, at about a 10 ns delay, direct temporal overlap becomes negligible, and the ASE‐to‐PL ratio reaches its minimum. This regime represents a transition from overlap‐dominated dynamics to relaxation processes occurring between the two excitations. Both residual carrier populations and an elevated local temperature may influence the response of the second excitation pulse. For delays exceeding 10 ns, the pulses are temporally separated, removing the contribution from increased instantaneous excitation intensity. With increasing delay, carrier populations progressively relax, while slower thermal relaxation may continue to influence the gain medium. Consequently, the gradual recovery of ASE at longer delays is consistent with an increasing contribution from thermal relaxation. At delays longer than approximately 1 µs, the emission approaches the response observed under single‐pulse excitation, indicating substantial relaxation of the photoexcited system between consecutive pulses. We further emphasize that the characteristic timescales determined from these measurements are specific to the experimental conditions at 230 K and are not quantitatively extrapolated to the long‐pulse measurements described below.

In addition, to demonstrate lasing effects, we used commercially available 50 µm silica microspheres that act as circular optical cavities, where emitted light circulates along the sphere's circumference via total internal reflection (Figure S5a). These cavities support resonant modes known as whispering‐gallery modes (WGMs) [33]. Using this configuration, we observe well‐resolved, stable lasing modes with a clear threshold‐like temperature dependence (Figure S5b–d). All spectra were collected from a single microsphere to ensure consistency.

To further analyze the cavity properties, we averaged the emission spectrum and expressed it as a function of wave vector in k‐space (Figure S5e). Fourier transformation of this spectrum yields the corresponding optical path‐length distribution (Figure S5e, inset), revealing a dominant mean optical path length of 48 µm, in good agreement with the microsphere size range. For CsPbBr_3_ films investigated in this work, the lasing modes exhibit a highly stochastic spectral distribution (Figure S5c,f), reflecting the disordered nature of optical coupling between the thin‐film gain medium and the microsphere cavity.

### 2.3 Role of Thermal Management in Long‐Pulse and CW‐Pumped ASE

Considering the impact of long‐pulse excitation on ASE behavior, we investigated emission properties from two similar CsPbBr_3_ films deposited on different substrates: soda‐lime glass and sapphire. These substrates differ greatly in thermal conductivity at room temperature, with soda‐lime glass at approximately 1 W m^−1^ K^−1^ and sapphire at 40 W m^−1^ K^−1^ [34, 35]. This comparison enables the identification of the contribution of thermally driven effects to optical gain. Importantly, both films were deposited simultaneously within the same evaporation batch, ensuring the same composition and thickness and minimizing deposition‐related sample‐to‐sample variations.

To evaluate possible substrate‐induced differences, we compared the morphology and initial optical properties of both films (Figure S1). AFM measurements reveal similarly smooth, compact morphologies, with RMS roughness values of ∼2 and ∼1 nm and average grain sizes of ∼90 and ∼80 nm for the films on soda‐lime glass and sapphire, respectively. Additionally, the absorption spectra are nearly identical, indicating comparable optical absorption and band‐edge characteristics, while the PL spectra exhibit similar profiles with only an approximately 3 nm difference in peak position (Figure S6a,b). This small spectral offset is not attributed to thermal‐management effects, as light‐induced heating is expected to be negligible under the femtosecond excitation conditions used for these measurements.

The initial gain characteristics were further compared by measuring the ASE thresholds at room temperature under 450 nm femtosecond excitation. Both films exhibit comparable low‐threshold ASE, of approximately 3.2 µJ cm^−2^ on soda‐lime glass and 2.5 µJ cm^−2^ on sapphire (Figure S6c–e). In contrast to these relatively small differences under short‐pulse excitation, a pronounced divergence in ASE behavior emerges with increasing excitation pulse duration.

For the CsPbBr_3_ film deposited on soda‐lime glass, stable ASE can be sustained only up to pulse widths of approximately 2 µs. Meanwhile, the film deposited on sapphire exhibits stable ASE for excitation pulse widths up to 30 µs (Figure 3a,b). Considering the comparable initial morphology and optical‐gain characteristics of the two films, together with the large difference in substrate thermal conductivity, this pronounced difference supports heat dissipation as the dominant factor governing their different long‐pulse ASE stability. Nevertheless, possible contributions from substrate‐dependent optical confinement and film‐substrate interfacial effects cannot be completely excluded.

**FIGURE 3:**
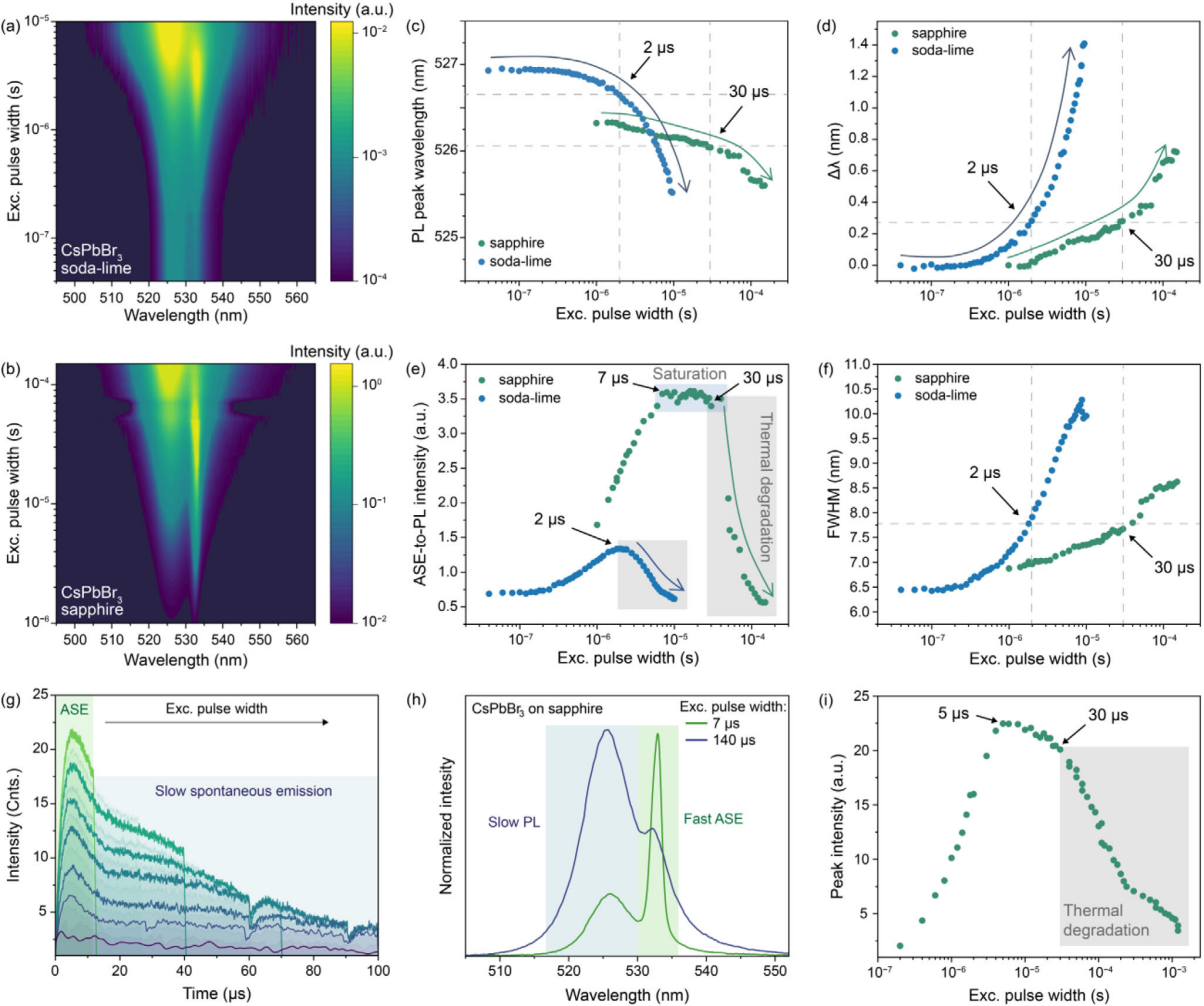
Light‐induced thermal degradation of ASE under long‐pulsed (quasi‐CW) excitation from thermally evaporated CsPbBr_3_ 100 nm‐thin films. (a,b) Emission spectra of the corresponding sample on soda‐lime (a) and sapphire (b) substrates under various pulse‐width excitations. (c–f) PL peak wavelength (c), shift of the PL peak wavelength (Δλ) (d), ASE‐to‐PL intensity ratio (e), and FWHM (f) from the corresponding samples (on soda‐lime glass—blue markers; on sapphire—green markers) under various pulse‐width excitations. (g) Time‐resolved PL decays from the thermally evaporated film on the sapphire substrate under various pulse‐width excitations, where a short, intense ASE lifetime and long PL decay are shown. (h) Emission spectra of the sample under 7 µs (green curve) and 140 µs (blue curve) pulsed excitations with an ASE band at 533.4 nm. (i) Peak intensity value vs. pulse‐width excitation from the experimental results presented in (g). All measurements were performed at 90 K in a nitrogen cryochamber under 450 nm laser excitation at 500 Hz, with a peak power of 10 W and an excitation area of 130 × 35 µm^2^.

By plotting the PL peak wavelength, PL peak shift, ASE‐to‐PL intensity ratio, and FWHM as functions of the excitation pulse width, we observe a clear correlation among all parameters for both samples (Figure 3c–f). For the film deposited on soda‐lime glass, ASE remains stable only up to pulse widths of approximately 2 µs; beyond this, thermal accumulation degrades the optical gain. In contrast, the sapphire‐supported film exhibits a quasi‐steady‐state optical response, with stable ASE maintained from about 7 µs to 30 µs, indicating a better balance between heat generation and dissipation. At pulse widths exceeding this value, thermal effects eventually dominate and suppress ASE.

To verify that these experimentally observed timescales are physically consistent with heat diffusion, we performed an order‐of‐magnitude transient thermal analysis (Note S1). For the 100‐nm CsPbBr_3_ film, the characteristic through‐film thermalization time is of the order of tens of nanoseconds. Thus, the performance of the film does not depend on the substrate for shorter (femtosecond or picosecond) pulses. Assuming that 30% of the incident optical pump is absorbed and that 50% of the absorbed energy is converted into heat, and given the high thermal diffusivity of sapphire, the estimated temperature rise of the CsPbBr_3_ film on sapphire is of the order of 23 K at 10 ns and approximately 30–180 K in the 1–30 µs regime. In contrast, the glass‐supported film enters a strong‐heating regime already on the microsecond timescale. These estimates are consistent with the experimentally observed onset of thermal effects and the substantially longer persistence of ASE on sapphire.

While quasi‐CW excitation mimics continuous carrier injection on electronic timescales, it allows partial thermal relaxation, whereas true CW excitation imposes sustained thermal load and represents the most stringent condition for optical gain. By analyzing the time‐resolved emission dynamics under excitation with short‐ to long‐pulse durations, we consistently resolve two decay components: a fast component associated with the ASE band and a slower component corresponding to the spontaneous emission band (Figure 3g,h). Under long‐pulse excitation (on the sapphire substrate), the ASE‐related emission is sustained up to ca. 12 µs. Notably, the optical response approaches a quasi‐steady‐state regime after about 5 µs of excitation, when the ASE efficiency is optimal for this sample, suggesting a transient balance between carrier generation, recombination, and heat dissipation (Figure 3i). Meanwhile, the pronounced ASE band is observed only for an excitation duration of approximately 30 µs. It indicates a balance between carrier injection and heat dissipation. Beyond this point, the ASE collapses due to continuous carrier generation and cumulative thermal effects, which suppress population inversion. Moreover, for pulses longer than ∼100 µs, irreversible degradation processes occur. Based on these results, extending excitation to longer pulse durations requires a focus on thermal management. Beyond substrate engineering, alternative approaches could suppress thermal accumulation in perovskite gain media, including integrating thermally conductive heat‐spreading layers, optimizing thermal contacts at film interfaces, and using device geometries that promote efficient heat dissipation [36, 37, 38]. Reducing nonradiative recombination may further improve thermal management by limiting the conversion of absorbed excitation energy into heat. Nevertheless, substrate‐based thermal management offers a comparatively simple approach to improve heat extraction without directly modifying the composition or intrinsic optical properties of the perovskite gain medium.

To clarify the temperature‐dependent properties of thermally evaporated CsPbBr_3_ films, we performed detailed measurements of absorption, PL, ASE, and random lasing from RT down to cryogenic temperatures (Figure S7). Across the entire temperature range, the absorption tail and the PL emission band overlap, indicating strong self‐absorption, while the ASE remains redshifted relative to the absorption tail (Figures S7d and S8a). This effect indicates that reabsorption remains a relevant loss mechanism even at low temperatures; meanwhile, it explains the stable gain observed in our samples: redshifted ASE indicates less heating, lower losses, and higher efficiency due to reduced reabsorption.

Notably, under conditions where ASE or random lasing is observed, the PL peak wavelength is systematically blueshifted compared with spectra of pure spontaneous emission (measured at considerably lower excitation intensities), particularly at temperatures below 180 K. This shift can be attributed to state‐filling and thermal effects at high excitation densities (Figure S8a,b). In this regime, the ASE intensity becomes more pronounced at reduced temperatures due to suppressed phonon‐assisted nonradiative recombination. Additionally, for samples exhibiting ASE or random lasing, the PL band FWHM is consistently larger than in samples showing only spontaneous emission, which may indicate increased thermal effects (Figure S8c).

Furthermore, time‐resolved PL measurements reveal that the mean carrier lifetime in thermally evaporated CsPbBr_3_ films increases with decreasing temperature across the PL emission range (Figures S8d and S9). At temperatures <100 K, long‐lived defect‐related recombination, reduced phonon scattering, and suppressed nonradiative recombination dominate, prolonging carrier lifetimes and thereby favoring the development of optical gain.

Overall, our results show that efficient heat dissipation, reduced reabsorption losses, and favorable carrier dynamics enable sustained optical gain and highlight the critical conditions needed to approach true CW operation.

## 3 Conclusion

In this work, we have systematically investigated ASE under long‐pulse and CW excitation in thermally evaporated CsPbBr_3_ thin films, revealing the fundamental thermal limitations governing sustained optical gain in perovskites. We show that short‐pulse excitation readily supports stable ASE because of minimal heat accumulation, whereas long‐pulse and CW excitation are fundamentally constrained by light‐induced thermal buildup, which enhances nonradiative recombination, suppresses population inversion, and ultimately degrades ASE. By combining time‐resolved spectroscopy, temperature‐dependent optical measurements, substrate engineering, and controlled excitation, we establish a comprehensive physical framework linking excitation duration, thermal dissipation, and gain stability. Through quantitative analysis, we identify excitation‐time thresholds and optimal operating regimes for thermally evaporated CsPbBr_3_ films, showing that stable ASE can be sustained for pulse widths up to 30 µs on high‐thermal‐conductivity sapphire substrates without encapsulation or post‐processing. These results establish that thermal management, rather than intrinsic gain limitations, is the dominant factor restricting long‐pulse and CW ASE in perovskite thin films, providing guidelines for quantitative analysis of thermal effects in perovskite gain media and representing a significant step toward developing electrically pumped and CW‐operated perovskite laser diodes.

## 4 Experimental Section

### 4.1 Fabrication of Single‐Source Thermally Evaporated CsPbBr_3_ Films

The CsPbBr_3_ thin films were fabricated by single‐source thermal evaporation of a melt‐grown precursor, as described in detail in our previous work [8].

### 4.2 Scanning Electron Microscopy (SEM)

Images were obtained using a scanning electron microscope (TFS Quanta 200F, Thermo Fisher Scientific) at an acceleration voltage of 10 kV in backscattered electron detection mode.

### 4.3 Atomic Force Microscopy (AFM)

High‐resolution images were obtained using an AFM (NX10 EFM, Park) in non‐contact mode (NCM) in the phase and z‐height regimes with SmartScan software. The scan rate was 0.3 Hz. The scan resolution was 526×526 pixels.

### 4.4 Absorption Spectra

UV–vis absorbance spectra were recorded using a spectrophotometer (V670, Jasco) equipped with an integrating sphere (ILN‐725, Jasco), a deuterium lamp for the UV region (employed at wavelengths below 330 nm), and a halogen lamp for the UV–vis–NIR region (employed at wavelengths above 330 nm). Temperature‐dependent optical absorption spectra were recorded using a white‐light deuterium source (SLS204/M, Thorlabs), a multimode fiber (M114L01; Thorlabs), a collimator (CVH100‐COL and LA4647; Thorlabs), and a diaphragm to restrict the probe spot size to ∼2 mm. The light transmitted through the sample was collected with a fiber coupler (CVH100‐COL and LA4647, Thorlabs) and guided to a broadband detector (QE Pro spectrometer, Ocean Insight) through an optical fiber (M113L01, Thorlabs). The thermally evaporated CsPbBr_3_ film deposited on a quartz substrate (PGO) was mounted in a liquid‐helium cryostat (ARS DE204AE) using heat‐sink paste (Dow Corning), leaving a small sample area without paste for transmission measurements. Energy‐dependent optical‐density spectra 𝑂𝐷(𝐸) were obtained from the transmitted light intensity according to 𝑂𝐷(𝐸)  =  −𝑙𝑜𝑔_10_[(𝐼𝑠_𝑎𝑚𝑝𝑙𝑒_(𝐸) − 𝐼_𝑑𝑎𝑟𝑘_)/(𝐼_𝑠𝑢𝑏𝑠𝑡𝑟𝑎𝑡𝑒_(𝐸) − 𝐼_𝑑𝑎𝑟𝑘_)], where 𝐼_𝑠𝑎𝑚𝑝𝑙𝑒_(𝐸) and 𝐼_𝑠𝑢𝑏𝑠𝑡𝑟𝑎𝑡𝑒_(𝐸) are the recorded intensities after passing through the sample and substrate only, respectively, and 𝐼_𝑑𝑎𝑟𝑘_ are the spectrally constant dark counts. Since light scattering and specular reflection are comparably weak, the optical‐density spectra (𝐸) approximately correspond to the absorbance spectra of the thin film. The spectral measurement was repeated at various temperatures employing a heating rate of 2 K per minute.

### 4.5 Photoluminescence and Amplified Spontaneous Emission

The samples were excited using a tunable femtosecond laser system consisting of a pulsed laser (ORIGAMI XP, 250 fs pulse width, 10 kHz repetition rate) and an optical parametric amplifier (ORIGAMI IRO, NKT Photonics), nanosecond laser diodes (PLPT9 450LB_E, ams OSRAM, 447 nm, 10 ns), and an A40640 dual‐beam laser module (NEJE, 450 nm, 12 W) coupled with a TTL module and a waveform generator (SDG1032X, Siglent) for repetition rate and pulse width control. Temperature‐dependent measurements were conducted under a nitrogen atmosphere in a probe stage (HFS600E‐PB4, Linkam). The laser pulse energy was measured using a laser power/energy meter (Centauri, MKS Ophir) equipped with a µJ‐measuring head (PE9‐ES‐C and PE9‐C, MKS Ophir). A variable metallic neutral‐density filter attenuated the laser beam intensity. The fs‐laser beam was focused into a tight stripe (~ 0.9×4.4 mm) using a cylindrical lens with a focal length of 75 mm. The sample's optical emission was collected over a relatively large solid angle, inclined at about 45° to the sample surface, and focused into a multimode optical fiber. It was then recorded via a broadband spectrometer (CCS200/M, Thorlabs). A long‐pass filter in the emission path blocked Rayleigh‐scattered excitation light. For the streak‐camera measurements (Figure 1d), the instrument response function (IRF) of the setup was determined by measuring the reflected excitation pulse from the sample using the streak camera (C10910–05, Hamamatsu Photonics) with a 2 ns time range and a 150 lines mm^−1^ grating. The excitation was provided by a tunable femtosecond laser system (ORIGAMI XP and ORIGAMI IRO, NKT Photonics) operating at 430 nm with a pulse duration of 250 fs and a repetition rate of 50 kHz. The measured temporal profile was background‐corrected and fitted with a Gaussian function, yielding an IRF of 2.4 ps (Figure S10). For the double‐pulse measurements (Figure 2d–f), the temporal profiles of the two excitation pulses were characterized using a high‐speed free‐space detector (DET025AL, Thorlabs) and a digital oscilloscope (DPO 2024B, Tektronix). Gaussian fits to the measured temporal profiles yielded pulse widths (FWHM) of 11.1 and 10.7 ns for the two laser diodes, respectively (Figure S11). The relative temporal delay between the pulses was electronically controlled with a timing resolution of 1 ns, corresponding to an estimated delay uncertainty of approximately ±0.5 ns. Emission spectra were recorded using a CCS200/M spectrometer with an integration time of 100 ms. The excitation lasers operated at a repetition rate of 200 Hz (5 ms), resulting in approximately 20 excitation events per recorded spectrum.

### 4.6 Time‐Resolved Photoluminescence Decay and Lifetime Measurements

The time‐ and wavelength‐resolved images were recorded under ambient conditions using a streak camera (C10910‐05, Hamamatsu Photonics). The excitation source was a tunable femtosecond laser system (450 nm, 250 fs, 10 kHz, ORIGAMI XP and ORIGAMI IRO, NKT Photonics). The excitation‐pulse‐width‐dependent time‐resolved PL decays from the thermally evaporated films were recorded at 90 K under a nitrogen atmosphere using a high‐speed free‐space detector (DET025AL, Thorlabs). The excitation source was an A40640 dual‐beam laser module (NEJE, 450 nm, 12 W) coupled with a TTL module and a waveform generator (SDG1032X, Siglent).

### 4.7 Whispering‐Gallery‐Mode Lasing

The samples were excited using the nanosecond laser diode (PLPT9 450LB_E, ams OSRAM, 447 nm, 10 ns, 1 kHz, 180 nJ). The spectra were collected over a 45° solid angle to the sample surface, focused into a multimode optical fiber, and recorded by a monochromator coupled to a high‐resolution imaging spectrograph (Princeton Instruments). Spectra were measured with a grating 1800 (or 300) lines/mm. Temperature‐dependent measurements were conducted under a nitrogen atmosphere in a probe stage (HFS600E‐PB4, Linkam).

## Author Contributions

The manuscript was written through the contributions of all authors. All authors have approved the final version of the manuscript.

## Funding

This work was supported by the Swiss National Science Foundation (SNSF), Grant Agreement No. 10004265, SLIGHT‐WAVE, and ETH Zürich through the ETH+ Project SynMatLab: Laboratory for Multiscale Materials Synthesis.

## Conflicts of Interest

The authors declare no conflicts of interest.

## Supporting information




**Supporting File**: advs78165‐sup‐0001‐SuppMat.docx.

## Data Availability

The data that support the findings of this study are available from the corresponding authors upon request. The data are not publicly available due to privacy or ethical restrictions.

## References

[1] L. Protesescu , S. Yakunin , M. I. Bodnarchuk , et al., “Nanocrystals of Cesium Lead Halide Perovskites (CsPbX_3_, X = Cl, Br, and I): Novel Optoelectronic Materials Showing Bright Emission With Wide Color Gamut,” Nano Letters 15, no. 6 (2015): 3692–3696, 10.1021/nl5048779. PMC4462997 25633588

[2] Q. A. Akkerman , T. P. T. Nguyen , S. C. Boehme , et al., “Controlling the Nucleation and Growth Kinetics of Lead Halide Perovskite Quantum Dots,” Science 377, no. 6613 (2022): 1406–1412, 10.1126/science.abq3616. 36074820

[3] H. Cho , C. Wolf , J. S. Kim , et al., “High‐Efficiency Solution‐Processed Inorganic Metal Halide Perovskite Light‐Emitting Diodes,” Advanced Materials 29, no. 31 (2017): 1700579, 10.1002/adma.201700579. 28608541

[4] Y. Yang , M. Yang , D. T. Moore , et al., “Top and Bottom Surfaces Limit Carrier Lifetime in Lead Iodide Perovskite Films,” Nature Energy 2, no. 2 (2017): 16207, 10.1038/nenergy.2016.207.

[5] B. R. Sutherland and E. H. Sargent , “Perovskite Photonic Sources,” Nature Photonics 10, no. 5 (2016): 295–302, 10.1038/nphoton.2016.62.

[6] L. M. Herz , “Charge‐Carrier Mobilities in Metal Halide Perovskites: Fundamental Mechanisms and Limits,” ACS Energy Letters 2, no. 7 (2017): 1539–1548, 10.1021/acsenergylett.7b00276.

[7] H. Utzat , W. Sun , A. E. K. Kaplan , et al., “Coherent Single‐Photon Emission From Colloidal Lead Halide Perovskite Quantum Dots,” Science 363, no. 6431 (2019): 1068–1072, 10.1126/science.aau7392. 30792359

[8] Y. Kominko , S. Sabisch , A. Kanak , et al., “Stable, Room‐Temperature, Low‐Threshold Amplified Spontaneous Emission From Thermally Evaporated Cesium Lead Halide Perovskites,” ACS Nano 19, no. 32 (2025): 29216–29227, 10.1021/acsnano.5c03771. PMC12369004 40667683

[9] Y. Hassan , J. H. Park , M. L. Crawford , et al., “Ligand‐Engineered Bandgap Stability in Mixed‐Halide Perovskite LEDs,” Nature 591, no. 7848 (2021): 72–77, 10.1038/s41586-021-03217-8. 33658694

[10] Y.‐H. Kim , S. Kim , A. Kakekhani , et al., “Comprehensive Defect Suppression in Perovskite Nanocrystals for High‐Efficiency Light‐Emitting Diodes,” Nature Photonics 15, no. 2 (2021): 148–155, 10.1038/s41566-020-00732-4.

[11] S. Mathews , R. Ramesh , T. Venkatesan , and J. Benedetto , “Ferroelectric Field Effect Transistor Based on Epitaxial Perovskite Heterostructures,” Science 276, no. 5310 (1997): 238–240, 10.1126/science.276.5310.238. 9092468

[12] M. Liu , M. B. Johnston , and H. J. Snaith , “Efficient Planar Heterojunction Perovskite Solar Cells by Vapour Deposition,” Nature 501, no. 7467 (2013): 395–398, 10.1038/nature12509. 24025775

[13] L. Meng , E.‐P. Yao , Z. Hong , et al., “Pure Formamidinium‐Based Perovskite Light‐Emitting Diodes With High Efficiency and Low Driving Voltage,” Advanced Materials 29, no. 4 (2017): 27879016, 10.1002/adma.201603826. 27879016

[14] S. Yakunin , L. Protesescu , F. Krieg , et al., “Low‐Threshold Amplified Spontaneous Emission and Lasing From Colloidal Nanocrystals of Caesium Lead Halide Perovskites,” Nature Communications 6 (2015): 8056, 10.1038/ncomms9056. PMC4560790 26290056

[15] S. Feng , B. Povilus , and S. Yang , “Color Tuning Halide Perovskites: Optical Amplification and Lasing,” Materials Today Advances 20 (2023): 100431, 10.1016/j.mtadv.2023.100431.

[16] S. A. Veldhuis , P. P. Boix , N. Yantara , et al., “Perovskite Materials for Light‐Emitting Diodes and Lasers,” Advanced Materials 28, no. 32 (2016): 6804–6834, 10.1002/adma.201600669. 27214091

[17] R. Zhang , Z. Zhou , X. Li , et al., “Low‐Threshold and Ultrastable Amplified Spontaneous Emission From CsPbBr_3_@Glass via Glass Network Modulation,” ACS Nano 19, no. 14 (2025): 14318–14329, 10.1021/acsnano.5c01653. 40192026

[18] C. Zhou , Y. Zhong , H. Dong , et al., “Cooperative Excitonic Quantum Ensemble in Perovskite‐assembly Superlattice Microcavities,” Nature Communications 11 (2020): 329, 10.1038/s41467-019-14078-1. PMC6965136 31949149

[19] P. Brenner , O. Bar‐On , M. Jakoby , et al., “Continuous Wave Amplified Spontaneous Emission in Phase‐Stable Lead Halide Perovskites,” Nature Communications 10, no. 1 (2019): 988, 10.1038/s41467-019-08929-0. PMC6395683 30816111

[20] F. Zhao , A. Ren , P. Li , Y. Li , J. Wu , and Z. M. Wang , “Toward Continuous‐Wave Pumped Metal Halide Perovskite Lasers: Strategies and Challenges,” ACS Nano 16 (2022): 7116–7143, 10.1021/acsnano.1c11539. 35511058

[21] M. S. Alias , Z. Liu , A. Al‐atawi , T. K. Ng , T. Wu , and B. S. Ooi , “Continuous‐Wave Optically Pumped Green Perovskite Vertical‐cavity Surface‐emitter,” Optics Letters 42, no. 18 (2017): 3618–3621, 10.1364/OL.42.003618. 28914916

[22] Z. Huang , Y. Ren , Y. Wu , et al., “Sustained Continuous‐Wave Lasing in Quantum Dot Microfluids,” Advanced Materials 37, no. 42 (2025): 09457, 10.1002/adma.202509457. 40767053

[23] Y. Li , H. Hu , A. Farag , et al., “Enhancement of Amplified Spontaneous Emission by Electric Field in CsPbBr3 Perovskites,” Nano Letters 23, no. 5 (2023): 1637–1644, 10.1021/acs.nanolett.2c02944. PMC9999453 36852434

[24] X. Zhang , Y. Li , G. He , et al., “Quasi‐CW Amplified Spontaneous Emission in Air‐Processed Quasi‐2D Perovskite Thin Films With High Stability,” Advanced Functional Materials 34, no. 23 (2024): 2315172, 10.1002/adfm.202315172.

[25] A. Gharajeh , R. Haroldson , Z. Li , et al., “Continuous‐wave Operation in Directly Patterned Perovskite Distributed Feedback Light Source at Room Temperature,” Optics Letters 43, no. 3 (2018): 611–614, 10.1364/OL.43.000611. 29400853

[26] I. Suárez , “Stimulated Emission Mechanisms in Perovskite semiconductors,” in Photonic Materials and Applications, Eds.: J. P. Martínez‐Pastor and P. P. Boix (Elsevier, 2023).

[27] S. Milanese , M. L. D. Giorgi , M. Anni , M. I. Bodnarchuk , and L. Cerdán , “Rationalizing the Amplified Spontaneous Emission Mechanism in CsPbBr_3_ Perovskite Nanocrystals Films by Means of Optical Gain Measurements,” Advanced Optical Materials 12, no. 27 (2024): 2401078, 10.1002/adom.202401078.

[28] W. Zhao , Z. Qin , C. Zhang , et al., “Optical Gain From Biexcitons in CsPbBr_3_ Nanocrystals Revealed by Two‐dimensional Electronic Spectroscopy,” The Journal of Physical Chemistry Letters 10, no. 6 (2019): 1251–1258, 10.1021/acs.jpclett.9b00524. 30811208

[29] A. Barfüßer , J. Feldmann , and Q. A. Akkerman , “Biexcitonic Optical Gain in CsPbBr_3_ Quantum Dots,” ACS Photonics 11, no. 12 (2024): 5350–5357, 10.1021/acsphotonics.4c01659.

[30] D. P. Strandell and P. Kambhampati , “Observing Strongly Confined Multiexcitons in Bulk‐Like CsPbBr_3_ Nanocrystals,” The Journal of Chemical Physics 158, no. 15 (2023): 154702, 10.1063/5.0142113. 37093993

[31] M. N. Ashner , K. E. Shulenberger , F. Krieg , et al., “Size‐Dependent Biexciton Spectrum in CsPbBr_3_ Perovskite Nanocrystals,” ACS Energy Letters 4, no. 11 (2019): 2639–2645, 10.1021/acsenergylett.9b02041.

[32] B. Li , H. Huang , G. Zhang , et al., “Excitons and Biexciton Dynamics in Single CsPbBr_3_ Perovskite Quantum Dots,” The Journal of Physical Chemistry Letters 9, no. 24 (2018): 6934–6940, 10.1021/acs.jpclett.8b03098. 30484306

[33] M. R. Foreman , J. D. Swaim , and F. Vollmer , “Whispering Gallery Mode Sensors,” Adv Opt Photon 7, no. 2 (2015): 168–240, 10.1364/AOP.7.000168. PMC4786191 26973759

[34] L. P. Janssen and M. M. Warmoeskerken , Transport Phenomena Data Companion (VSSD, 2006).

[35] Kyocera Corporation , Single‐Crystal Sapphire (Al_2_O_3_) Property.

[36] L. Zhao , K. Roh , S. Kacmoli , et al., “Thermal Management Enables Bright and Stable Perovskite Light‐Emitting Diodes,” Advanced Materials 32, no. 25 (2020): 2000752, 10.1002/adma.202000752. 32406172

[37] G. Li , Z. Hou , Y. Wei , et al., “Efficient Heat Dissipation Perovskite Lasers Using a High‐Thermal‐Conductivity Diamond Substrate,” Science China Materials 66 (2023): 2400–2407, 10.1007/s40843-022-2355-6.

[38] T. Haeger , R. Heiderhoff , and T. Riedl , “Thermal Properties of Metal‐Halide Perovskites,” Journal of Materials Chemistry C 8, no. 41 (2020): 14289–14311, 10.1039/d0tc03754k.

