# Supplementary material for: CW‐Pumped Amplified Spontaneous Emission From Thermally Evaporated CsPbBr3 Films
Source: Adv Sci (Weinh). 2026 Oct 11:e78165. Online ahead of print. doi: 10.1002/advs.78165 (PMC13655262; doi:10.1002/advs.78165)
Supplement: Supplementary file 1 [file ADVS-9999-e78165-s001.docx]

Supporting Information

CW-Pumped Amplified Spontaneous Emission from Thermally Evaporated CsPbBr_3_ Films

Yuliia Kominko, Willem Verheijen, Gabriele Rainò, Simon C. Boehme, Maksym V. Kovalenko*, and Sergii Yakunin*

**Figure** **S1**. AFM images of the 100-nm-thick thermally evaporated CsPbBr_3_ films on soda-lime glass (a-b) and sapphire (c-d) substrates scanned in NCM phase (a,c) and z-height (b,d) scan regimes, respectively.

**Figure S2**. a) TRPL curves of the 100-nm CsPbBr_3_ evaporated film recorded at 525 nm under different excitation intensities of a 355 nm picosecond laser with a 10 MHz repetition rate. b) Mean lifetime values (emission amplitude-weighted) *vs.* excitation intensity.

**Figure S3**. Time- and wavelength-resolved streak camera images from a 100-nm-thick thermally evaporated CsPbBr_3_ film. **a)** PL spectra of the early-time emission (t < 30 ps) under various excitation energies. **b)** Normalized time-resolved PL traces in the PL region (spectrally integrated between 528 and 532 nm) and ASE region (spectrally integrated between 540 and 544nm) under 6 nJ and 8 nJ laser excitation energies. **c)** Time- and wavelength-resolved streak camera images under 1 nJ, 2 nJ, 4 nJ, 6 nJ, and 8 nJ laser excitation energies. All spectra were recorded under 450 nm fs-laser excitation with a 10 kHz repetition rate at RT.

**Figure S4.** SEM image and energy-dispersive spectroscopy (EDS) of the 100 nm-thick thermally evaporated CsPbBr_3_ films reveal recrystallization of the nanocrystalline domains under CW laser excitation, driven by the light-induced temperature increase, without a change in stoichiometry (considering a measurement error of 5%).

**Figure S5.** Whispering-gallery-mode lasing from CsPbBr_3_. **a)** Schematic image of WGM lasing in a 50 µm silica sphere resonator, covered by the thermally evaporated CsPbBr_3_ film on a sapphire substrate. **b-c)** Evolution of the PL spectra from the corresponding sample under 2.8×10^3^ laser pulses with a 1 kHz repetition rate, showing the operational stability of the emission. Inset: photo of the 50 µm silica sphere under laser beam excitation. **d)** Temperature-dependent PL spectra of WGM lasing under constant excitation conditions. **e)** Fast Fourier transform (FFT) of the averaged data from (b), together with the subtracted data by the fitted line, showing a peak value of 48.1 µm. **f)** Evolution of the FFT of (b). All spectra were excited at 450 nm using 10 ns laser pulses at a 1 kHz repetition rate, with 180 nJ of excitation energy, under a nitrogen atmosphere at 170 K (except for temperature-dependent spectra).

**Figure S6**. Optical characterization of a 100 nm-thick thermally evaporated CsPbBr_3_ thin film on soda-lime glass and sapphire substrates. **a)** Optical absorption spectra of the corresponding samples. **b)** Emission spectra of the corresponding samples with fs-laser excitation at 450 nm, 10 kHz repetition rate, and 2 μJ cm^–2^ energy density at RT. **c-d)** Emission spectra of evaporated CsPbBr_3_ film on soda-lime glass (c) and sapphire (d) substrates at different excitation energies of the fs-laser at 450 nm, 10 kHz repetition rate. **e)** Emission intensity *vs.* excitation energy at the emission wavelengths of the ASE band from the sample on soda-lime glass (blue markers) and sapphire (green markers), respectively. The ASE thresholds were determined as *ca.* 3.2 μJ cm^–2^ for the soda-lime and 2.5 μJ cm^–2^ for the sapphire by the intercept of the linear fits (blue and green lines) below and above the threshold.

**Figure S7.** Temperature-dependent optical properties from thermally evaporated CsPbBr_3_ films. **a)** Temperature-dependent absorbance of the corresponding sample. **b)** Temperature-dependent PL under 450 nm laser excitation. **c)** Temperature- and wavelength-resolved mean lifetime of emission from the corresponding sample together with the PL spectra at 300 K (black curve) and 50 K (white curve). **d-e)** Temperature-dependent PL with ASE band (d) and random lasing (e) features under 10 ns laser excitation of the sample on a soda-lime substrate. Inset in (d): temperature-dependent absorption peak and absorption tail at their 7.5% values. **f)** Emission spectra of the evaporated film on a sapphire substrate at different excitation powers of the 450-nm CW laser at 90 K.

**Figure S8.** Analysis of temperature-dependent optical properties presented in Figure S7. **a)** Temperature-dependent wavelength of the PL peak in the case of emission without gain (below the ASE threshold, blue marks), emission with the presence of random lasing (green marks), and ASE (red marks), together with the temperature-dependent absorption peak and absorption tail at their 10% values. **b)** Temperature-dependent PL-to-ASE (random lasing) intensity ratio showing its exponential behavior and T_0_ near 33 K. **c)** Temperature-dependent FWHM of the PL band in the case of emission without gain (below the ASE threshold, blue marks), emission with the presence of random lasing (green marks), and ASE (red marks), showing light-induced heating of the material under high excitation required for the stimulated emission. **d)** Wavelength-resolved mean lifetime of emission from the thermally evaporated film at different temperatures (detailed data presented below in Figure S9).

**Figure S9.** Time-resolved emission spectra from the thermally evaporated CsPbBr_3_ film at various temperatures.

**Figure S10. a)** Streak camera image of the reflected light of a femtosecond laser to determine the instrument response function. **b)** Temporal response obtained from the streak camera image in (a), showing a Gaussian-shaped instrument response with a FWHM of 2.4 ps.

**Figure S11.** The temporal profiles of the 10 ns laser diodes used as excitation sources for the double-pulse experiment in Figure 2d-f. Gaussian fits yield FWHM pulse widths of 11.1 ns and 10.7 ns for laser diodes 1 and 2, respectively.

**Supplementary Note 1.** Basic quantitative analysis of transient heating.

To assess whether the characteristic timescales observed in the long-pulse experiments are physically consistent with heat diffusion, we performed an order-of-magnitude analysis of transient heating in the 100-nm CsPbBr_3_ film/substrate system. The calculation is not intended as direct thermometry. Rather, it estimates characteristic thermal diffusion lengths, response times, and local temperature rises under the experimental excitation conditions. The largest uncertainty is the fraction of incident optical power ultimately converted into heat.

*Experimental parameters and heat-generation estimate*

For consistency with the long-pulse measurements, the calculation uses a 450-nm pump with a peak optical power of 10 W focused to approximately 130 × 35 µm². The illuminated area is therefore *A* = 4.55 × 10^-9^ m^2^.

Approximately 30% of the incident 450-nm pump light is absorbed in the CsPbBr_3_ film. We assume that 50% of this absorbed optical energy is converted into heat during the ASE process, with the remainder leaving the excitation volume primarily through ASE emission. The effective fraction of incident pump power converted into heat is therefore *η_h_* = 0.15.

*Thermal parameters*

**Table SN1.** Thermal parameters used for thermal analysis.

| Material | α [m^2^ s^-1^] | c [J kg^-1^ K^-1^] | ρ [kg m^-3^] |
| --- | --- | --- | --- |
| CsPbBr3 film^[s1]^ | 3.0 × 10^-7^ | 300 | 4700 |
| Sapphire^[S2–S3]^ | 6.6 × 10^-4^ | 95 | 4000 |
| Soda-lime glass^[S4–S5]^ | ~7.3 × 10^-7^ | 780 | 2500 |

*Characteristic thermal diffusion times and lengths*

For a 100-nm CsPbBr_3_ film, the characteristic time for heat to diffuse through the film is: *τ_f_* ≈ *d*²/α ≈ 30 ns. Thus, the 10-ns regime is comparable to the through-film thermalization time. On the microsecond timescale, heat flow into and within the substrate mainly governs the thermal response. The thermal diffusion length is estimated as *L_diff_* = (αt)^0.5^.

**Table SN2.** Estimated thermal diffusion lengths *L_diff_*.

| Pulse duration | CsPbBr_3_ film [µm] | Sapphire [µm] | Soda-lime glass [µm] |
| --- | --- | --- | --- |
| 10 ns | 0.06 | 3 | 0.09 |
| 1 µs | 0.6 | 30 | 0.9 |
| 5 µs | 1.2 | 60 | 1.9 |
| 30 µs | 3 | 140 | 4.7 |
| 10 ns | 0.06 | 3 | 0.09 |

The pump spot has dimensions 130 × 35 µm^2^. In sapphire, the thermal diffusion length is already comparable to the narrow spot size on a sub-microsecond timescale and approaches the long spot size after several microseconds. This is consistent with a crossover toward efficient finite-spot heat spreading on the few-microsecond timescale. In glass, the diffusion length remains only several micrometers even after 30 µs, so the deposited heat remains strongly localized beneath the pumped region.

*Estimated temperature rise of the CsPbBr_3_ gain layer*

At 10 ns, the pulse duration is shorter than the characteristic through-film diffusion time. An upper-limit estimate can therefore be obtained by treating the film as approximately adiabatic.

*ΔT_ad_* ≈ *η_h_* *E*/(*cρAd*) ≈ 23 K	(S1)

Thus, the initial 10-ns thermal excursion of the gain layer is expected to be on the order of 20–25 K and should depend only weakly on the substrate because heat has not yet fully crossed the film.

For pulses with duration t ≫ *τ_f_*, the film becomes approximately equilibrated through its thickness. For uniform heat transfer, the mean substrate temperature just below the film/substrate interface is approximately *ΔT*_film−int_ ≈ *η_h_* *E*/(*c_substr_ρ_substr_A L_diff_*).	(S2)

Adding this approximate through-film temperature difference to the calculated substrate-interface temperature gives the following order-of-magnitude local temperature rises in the optically excited CsPbBr_3_ layer.

**Table SN3**. Local CsPbBr_3_ temperature rise.

| Pulse duration | CsPbBr3@sapphire ΔT [K] | CsPbBr3@glass ΔT [K] | Interpretation/model validity |
| --- | --- | --- | --- |
| 10 ns | 23 | 23 | Approximate adiabatic film estimates; substrate contribution is still limited |
| 1 µs | 34 | 200 | Order-of-magnitude estimate considering heat spread in the substrate. |
| 5 µs | 76 | 440 |  |
| 30 µs | 185 | 1100 |  |

**Supplementary References:**

S1. Haeger, T.; Wilmes, M.; Heiderhoff, R.; Riedl, T. "Simultaneous Mapping of Thermal Conductivity, Thermal Diffusivity, and Volumetric Heat Capacity of Halide Perovskite Thin Films: A Novel Nanoscopic Thermal Measurement Technique." *J. Phys. Chem. Lett.* 10 (2019), https://doi.org/10.1021/acs.jpclett.9b01053.

S2. Burghartz, St.; Schulz, B. "Thermophysical Properties of Sapphire, AlN and MgAl_2_O_4_ down to 70 K." *J. Nucl. Mater.* 212–215 (1994), https://doi.org/10.1016/0022-3115(94)90996-2.

S3. Ditmars, D. A.; Ishihara, S.; Chang, S. S.; Bernstein, G.; West, E. D. "Enthalpy and Heat-Capacity Standard Reference Material: Synthetic Sapphire (α-Al_2_O_3_) from 10 to 2250 K." *J. Res. Natl. Bur. Stand.* 87 (1982), https://doi.org/10.6028/jres.087.012.

S4. Baesso, M. L.; Shen, J.; Snook, R. D. "Time-resolved Thermal Lens Measurement of Thermal Diffusivity of Soda-Lime Glass." *Chem. Phys. Lett.* 197, (1992), https://doi.org/10.1016/0009-2614(92)85764-2.

S5. Astrath, N. G. C., Rohling, J. H., et al., "Time-resolved Thermal Lens Measurements of the Thermo-optical Properties of Glasses at Low Temperature down to 20 K." *Phys. Rev. B* 71 (2005), https://doi.org/10.1103/PhysRevB.71.214202.
